# Association between chronic obstructive pulmonary disease and in-hospital mortality after percutaneous coronary intervention: a retrospective cohort study in Germany

**DOI:** 10.1038/s41598-024-56255-3

**Published:** 2024-03-13

**Authors:** Nadine Hochhausen, Mare Mechelinck, Sebastian Billig, Rolf Rossaint, Felix Kork

**Affiliations:** Department of Anaesthesiology, Medical Faculty, RWTH Aachen University, Pauwelsstraße 30, 52074 Aachen, Germany

**Keywords:** Chronic obstructive pulmonary disease, Percutaneous coronary intervention, In-hospital mortality, Cardiovascular diseases, Respiratory tract diseases, Chronic obstructive pulmonary disease

## Abstract

Chronic obstructive pulmonary disease (COPD) is one of the leading chronic diseases worldwide. However, the impact of COPD on outcome after percutaneous coronary intervention (PCI) remains unclear. In this retrospective cohort study, we analyzed the data of hospitalized patients undergoing PCI in Germany between 2015 and 2019. We compared in-hospital mortality, hospital length of stay and peri-interventional ventilation time (VT) in patients with and without COPD, including different COPD severity grades, COPD with exacerbation (COPD_e_) and infection (COPD_i_). We analyzed the data of 3,464,369 cases undergoing PCI. A total of 291,707 patients (8.4%) suffered from COPD. Patients suffering from COPD died more often (2.4% vs. 2.0%; p < 0.001), stayed longer hospitalized (5 days (2–10) vs. 3 days (1–6); p < 0.001), were more frequent (7.2% vs. 3.2%) and longer ventilated (26 h (7–88) vs. 23 h (5–92); p < 0.001). Surprisingly, COPD was associated with a 0.78-fold odds of in-hospital mortality and with reduced VT (− 1.94 h, 95% CI, − 4.34 to 0.43). Mild to severe COPD was associated with a lower risk of in-hospital mortality and reduced VT, whereas very severe COPD, COPD_e_ and COPD_i_ showed a higher risk of in-hospital mortality. We found a paradoxical association between mild to severe COPD and in-hospital mortality, whereas very severe COPD, COPD_e_ and COPD_i_ were associated with higher in-hospital mortality. Further investigations should illuminate, whether comorbidities affect these associations.

## Introduction

Chronic obstructive pulmonary disease (COPD) is one of the world's leading chronic diseases. COPD presents itself with chronic respiratory symptoms, deterioration of lung function, ultimately leading to progressive impairment of health^1,2^. The incidence of perioperative complications in patients suffering from COPD is higher compared to the general population^3,4^. A study investigating the impact of COPD on postoperative mortality and complications in patients with lung resection for cancer showed that a reduced forced expiratory volume in 1 s (FEV1) predicts respiratory morbidity and mortality^5^. Another study demonstrated that COPD was associated with a higher risk for surgical site infections, sepsis, septic shock, pneumonia, readmission and mortality within 30 days after hip arthroplasties^6^. Patients with COPD suffer more often from adverse outcomes after thoracic, major abdominal^7^ and coronary artery bypass grafting surgery^8^.

Coronary artery disease (CAD) is also associated with a high mortality worldwide^9^. Due to the aging population and demographic shift, CAD and COPD will be common chronic diseases in the world`s population^10,11^. Since the risk factors of COPD are similar to those of CAD, it is reasonable that these two diseases often occur in association with each other^12,13^. The typical chronic inflammation in COPD may progress into a systemic inflammation, which may aggravate atherosclerosis^14,15^. A possible association between COPD and CAD has already been demonstrated^16^. Gold standard for the diagnosing and treating of CAD is percutaneous coronary intervention (PCI). However, the impact of COPD on patient’s outcome after PCI is discussed controversially. For example, it could be demonstrated that in mild to moderate COPD, cardiovascular diseases are one of the leading causes of mortality^17^. In addition, patients suffering from acute COPD exacerbation have an increased risk of cardiovascular events^18^ and patients with acute COPD exacerbation suffer more often from adverse outcomes after PCI^12^. On the other hand, some studies failed to demonstrate a negative impact of COPD on in-hospital major adverse cardiac outcomes^19^ or in-hospital mortality^20^. Against this contradicting background of evidence, the impact of COPD and COPD severity on outcome after PCI has not yet been investigated in sufficient detail. We aimed to shed more light on this question by analyzing a large cohort of patients undergoing PCI.

Therefore, we conducted a population-based retrospective cohort study to address this lack of studies. In this study, we investigated whether COPD, or grades of COPD severity, including COPD exacerbation (COPD_e_) and infection (COPD_i_), were associated with higher in-hospital mortality, hospital length of stay (HLOS) and peri-interventional ventilation time (VT) after PCI. The study population consisted of all hospitalized patients undergoing PCI in Germany between 2015 and 2019.

## Methods

### Patients and data source

We confirm that all methods were carried out in accordance with relevant guidelines and regulations. According to German Federal legislation, no institutional or review board approval as well as informed consent were necessary (Bundesstatistikgesetz; BStatG; Federal Statistic Law; https://www.gesetze-im-internet.de/bstatg_1987/index.html#BJNR004620987BJNE000607311).

The de-identified data was analyzed via controlled remote data processing: we designed an analysis protocol as a Stata do-file (Stata BE 17 for Windows, StataCorp, College Station, TX, USA) and tested it on sample data structure files provided by the Federal Statistical Office. Hereafter, the analysis of the Stata do-file was performed on the actual data (Stata 15 for Windows, StataCorp, College Station, TX, USA) by the Federal Statistical Office. The authors did not have access to the raw data. Results were returned to the authors after a detailed review and curation of the data to avoid any possible de-anonymization of individuals.

We conducted the retrospective cohort study using the German Diagnosis-Related Groups (G- DRG) Statistik (Source: RDC of the Federal Statistical Office and Statistical Offices of the Federal States, Source 10.21242/23141.2019.00.00.1.1.1, 10.21242/23141.2018.00.00.1.1.0, 10.21242/23141.2017.00.00.1.1.0, 10.21242/23141.2016.00.00.1.1.0, 10.21242/23141.2015.00.00.1.1.0, own calculations.) provided by the Federal Statistical Office of Germany (Statistisches Bundesamt, www.destatis.de). Here, all inpatient hospital cases in Germany accounted by case rates are registered in an annual survey. In Germany, the case based DRG accounting system is obligatory for hospitals. In addition to main and secondary diagnoses, sociodemographic characteristics are listed. We extracted all data from the previously mentioned data base such as age, sex, diagnoses, procedures, in-hospital mortality, HLOS and VT. We acquired the Charlson Comorbidity Index (CCI) with its items as described by Quan et al.^21^.

### Inclusion and exclusion criteria

**Figure 1:**
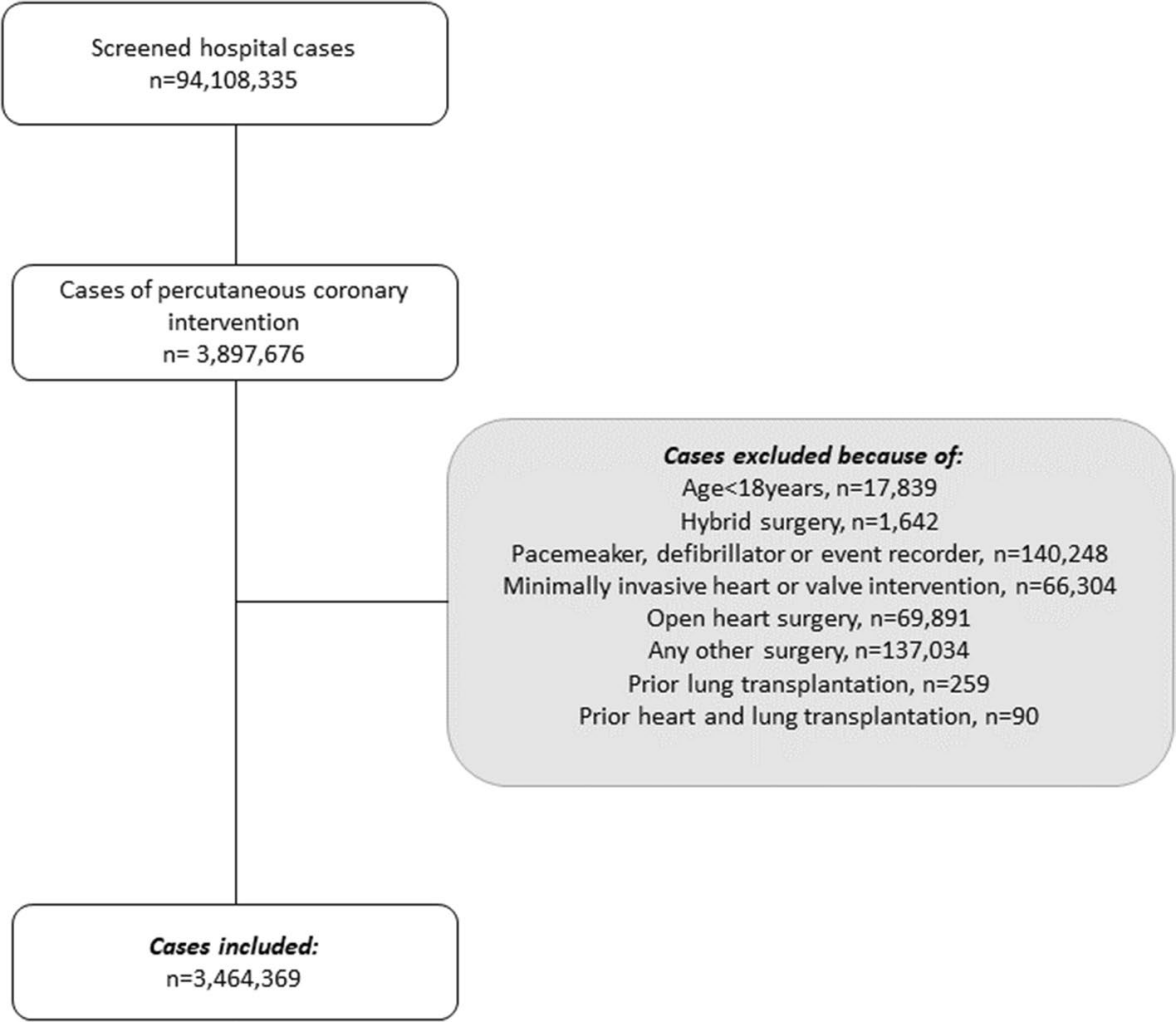
Flow chart of patient inclusion. Flow chart of patient inclusion of a population-based retrospective analysis investigating the impact of COPD and COPD severity on in-hospital mortality, hospital length of stay (HLOS) and ventilation time (VT) in 3,464,369 cases undergoing PCI.

We considered all patients undergoing PCI, regardless of the indication, between January 1, 2015, and December 31, 2019, in Germany eligible for inclusion. Patients with age under 18 years were excluded as well as hybrid surgery, implantation of a pacemaker, defibrillator or event recorder, minimally invasive heart or valve intervention, open heart surgery, any other surgery, prior lung transplantation and prior heart and lung transplantation (Fig. 1).

### Variables

We obtained the presence of COPD including the severity from hospital administrative data that save diagnoses in codes based on the International Statistical Classification of Diseases and Related Health Problems, Tenth Revision (ICD-10). A severity classification, based on airflow obstruction, is common using the Global Initiative for Chronic Obstructive Lung Disease classification (GOLD 1, 2, 3, and 4 or mild, moderate, severe, and very severe COPD). In particular, mild COPD is associated with a forced expiratory volume in 1 s (FEV_1_) of >  = 80% (GOLD 1), moderate COPD is associated with a FEV_1_ of 50–79% (GOLD 2), severe COPD is associated with a FEV_1_ of 30–49% (GOLD 3) and very severe COPD is associated with a FEV_1_ of < 30% (GOLD 4)^22^. However, this study is based on the ICD-10 severity classification of airway obstruction, which includes mild COPD defined by FEV_1_ of ≥ 70, moderate COPD defined by FEV_1_ of 50–70%, severe COPD defined by FEV_1_ of 35–50% and very severe COPD defined by FEV_1_ of < 35%. Moreover, another category was analyzed, namely unspecified COPD. This category is represented by an original code within the ICD-10 classification defined by the presence of COPD with an unspecified FEV_1._ In addition, we obtained the performance of PCI from the German procedures’ classification codes (Operationen- und Prozedurenschlüssel- OPS), a modified version of the International Classification of Procedures in Medicine (ICPM) as established by the World Health Organization (WHO). New categorical variables, such as comorbidities for calculating the CCI were created using ICD-10 diagnosis codes and OPS procedure codes (Additional file 1).

### Primary and secondary outcomes

The primary endpoint was in-hospital mortality, coded as a reason for discharge. The secondary endpoints were HLOS and VT.

### Statistical analyses

Since the study is a retrospective cohort study, no sample size calculation or power analysis was performed. The analysis protocol was coded by the authors and the Federal Statistical Office performed the analysis (Stata 15 for Windows, StataCorp, College Station, TX, USA).

Statistical significance was considered for p < 0.01. Frequencies were reported as numbers and percentages, continuous variables as median and interquartile ranges (IQR). Continuous variables were compared using the Mann–Whitney U-test, categorical variables using the chi-squared test. Different regression models were applied to estimate the association of COPD with in-house mortality, HLOS and VT.

Dependent variables were introduced in the regression models based on clinical relevance and availability in the database. Binary logistic regression models were fitted and cross-validated using the Stata module cvauroc (k = 10; robustness measure: area under the receiver operating curve (AUROC)) to estimate the association of COPD, including different COPD grades, and in-hospital mortality^23^. In addition, robust regression models were fitted and cross-validated using the Stata module ‘crossfold’ (k = 10; robustness measure: root mean square error (RMSE)) to estimate the association between COPD, including different grades of COPD severity, and HLOS and VT^24^.

### Ethics approval and consent to participate

No institutional or review board approval was required, since the included data were de-identified and accessed via controlled remote data processing without access to the actual data.

## Results

### Study population

The data set included 94,108,335 hospital cases between 2015 and 2019, of which 3,897,676 received PCI. Patients were excluded, if age was under 18 years (n = 17,839), additionally a pacemaker, defibrillator, or event recorder (n = 140,248) was in use. In addition, patients were excluded if hybrid surgery (n = 1642), minimally invasive heart or valve intervention (n = 66,304), open heart surgery (n = 69,891) or any other surgery (n = 137,034) was performed. Moreover, patients with prior lung transplantation (n = 259) or prior heart and lung transplantation (n = 90) were excluded. After exclusion of the aforementioned patients, data of 3,464,369 cases were analyzed (Fig. 1).

Table 1 summarizes the characteristics of the study cohort. The median age of all patients was 70 years (IQR, 60–78). 64.6% of the patients were male, and the Charlson Comorbidity Index (CCI) showed a median of 1 (IQR, 1–3). The most frequent comorbidities of all analyzed patients were congestive heart failure (37.0%), and myocardial infarction (31.8%).

**Table 1:** Characteristics and outcome parameters.

Characteristic	All patients (N = 3,464,369)	Patients with COPD (N = 291,707; 8.4%)	Patients without COPD (N = 3,172,662; 91.6%)	P-value COPD vs No-COPD
Sociodemographic characteristics				
Median age (IQR)—years	70 (60–78)	72 (64–78)	70 (59–78)	< 0.001
Sex—no. (%)				
Female—no (%)	1,225,560 (35.4)	104,058 (35.7)	1,121,502 (35.3)	P = 0.002
Male—no (%)	2,238,689 (64.6)	187,639 (64.3)	2,051,050 (64.7)	P = 0.002
Unknown—no (%)	120 (0.00)	10 (0.00)	110 (0.00)	P = 0.002
Median Charlson Comorbidity Index (IQR)—pts	1 (1–3)	3 (2–4)	1 (0–2)	< 0.001
Charlson Comorbidity Index Items—no. (%)				
Diabetes Mellitus				
Uncomplicated	746,758 (21.6)	70,337 (24.1)	676,421 (21.3)	< 0.001
With end-organ damage	181,482 (5.2)	24,176 (8.3)	157,306 (5.0)	< 0.001
Cancer				
Non-metastatic	36,182 (1.0)	4797 (1.6)	31,385 (1.0)	< 0.001
Metastatic	8090 (0.2)	1067 (0.4)	7023 (0.2)	< 0.001
Renal Disease	671,432 (19.4)	81,959 (28.1)	589,473 (18.6)	< 0.001
Congestive Heart Failure	1,282,091 (37.0)	151,566 (52.0)	1130,525 (35.6)	< 0.001
Chronic Pulmonary Disease	376,002 (10.9)	291,707 (100)	84,295 (2.7)	< 0.001
Peripheral Vascular Disease	342,789 (9.9)	47,557 (16.3)	295,232 (9.3)	< 0.001
Cerebrovascular Disease	148,777 (4.3)	15,931 (5.5)	132,846 (4.2)	< 0.001
Myocardial infarction	1,102,675 (31.8)	85,252 (29.2)	1017,423 (32.1)	< 0.001
Dementia	36,098 (1.0)	3784 (1.3)	32,314 (1.0)	< 0.001
Hemiplegia or paraplegia	36,004 (1.0)	3631 (1.2)	32,373 (1.0)	< 0.001
Liver disease				
Mild	46,700 (1.4)	6175 (2.1)	40,525 (1.3)	< 0.001
Moderate to severe	5213 (0.2)	608 (0.2)	4605 (0.2)	< 0.001
Rheumatoid disease	46,850 (1.4)	5372 (1.8)	41,478 (1.3)	< 0.001
Peptic ulcer disease	10,166 (0.3)	1179 (0.4)	8987 (0.3)	< 0.001
AIDS	3252 (0.1)	286 (0.1)	2966 (0.1)	0.442
Outcome parameter				
In-Hospital mortality—no. (%)	70,670 (2.0)	6,866 (2.4)	63,804 (2.0)	< 0.001
Hospital length of stay—d (IQR)	4 (1–7)	5 (2–10)	3 (1–6)	< 0.001
Peri-interventional ventilation time—h (IQR) No. of ventilation support (%)	24 (5–91) N = 122,911 (3.5)	26 (7–88) N = 20,933 (7.2)	23 (5–92) N = 101,978 (3.2)	< 0.001

Characteristics and outcome parameters of 3,464,369 hospitalized patients undergoing percutaneous coronary intervention.

The overall in-hospital mortality was 2.0%, the median HLOS was 4 days (IQR, 1–7) and the median VT was 24 h (IQR, 5–91) if ventilation support was needed (in 3.5%).

### Patients suffering from COPD

Out of 3,464,369 patients analyzed, a total of 291,707 patients (8.4%) suffered from COPD. Patients with COPD were older and of poorer health status related to CCI compared to patients without COPD. In COPD patients, the median age was 72 years (IQR, 64–78) and 64.3% were male. The CCI calculated in patients suffering from COPD was significantly higher compared to patients not suffering from COPD (CCI 3 (IQR, 2–4) vs. CCI 1 (IQR, 0–2), p < 0.001). The most frequent comorbidities in both groups were congestive heart failure (52.0% vs. 35.6%, p < 0.001) and myocardial infarction (29.2% vs. 32.1%, p < 0.001) (Table 1).

### Impact of COPD on in-hospital mortality, HLOS and VT

In-hospital mortality was higher in patients suffering from COPD (6,866 (2.4%) vs. 63,804 (2.0%), p < 0.001). Furthermore, patients with COPD stayed longer in the hospital (5 days (IQR, 2–10) vs. 3 days (IQR, 1–6), p < 0.001)). In addition, patients suffering from COPD needed more frequently ventilation support (7.2% vs. 3.2%) and presented a prolonged VT (26 h (IQR, 7–88) vs. 23 h (IQR, 5–92), p < 0.001)) compared to patients without COPD (Table 1).

### Impact of COPD severity on in-hospital mortality, HLOS and VT

Table 2 summarizes the characteristics of the study cohort categorized by COPD severity (mild to very severe COPD, GOLD 1–4).

**Table 2:** Characteristics categorized by COPD severity.

Characteristic	No COPD (N = 3,172,662/91.58%)	Mild COPD (GOLD 1) (N = 29,828/0.86%)	Moderate COPD (GOLD 2) (N = 46,903/1.35%)	Severe COPD (GOLD 3) (N = 30,055/0.87%)	Very severe COPD (GOLD 4) (N = 21,579/0.62%)	Unspecified COPD (N = 163,342/4.71%)
Sociodemographic characteristics						
Median age (IQR)—years	70 (59–78)	73 (64–79)	73 (64–79)	72 (64–78)	70 (63–76)	72 (64–79)
Sex—no. (%)						
Female—no (%)	1,121,502 (35.6)	XXX	XXX	XXX	7506 (34.8)	58,082 (35.6)
Male—no (%)	2,051,050 (64.7)	18,067 (60.6)	30,157 (64.3)	19,884 (66.2)	14,275 (66.2)	105,256 (64.4)
Unknown—no (%)	110 (0.0)	XXX	XXX	XXX	XXX	4 (0.0)
Median Charlson Comorbidity Index (IQR)—pts	1 (0–2)	3 (2–4)	3 (2–4)	3 (2–4)	3 (2–4)	3 (2–4)
Charlson comorbidity index items—no. (%)						
Diabetes Mellitus						
Uncomplicated	676,421 (21.3)	6883 (23.1)	10,867 (23.2)	6413 (21.3)	4229 (19.6)	41,945 (25.7)
With end-organ damage	157,306 (5.0)	2609 (8.8)	4305 (9.2)	2781 (9.3)	1691 (7.8)	12,790 (7.8)
Cancer						
Non-metastatic	31,385 (1.0)	517 (1.7)	932 (2.0)	652 (2.2)	445 (2.1)	2251 (1.4)
Metastatic	7023 (0.2)	97 (0.3)	209 (0.5)	175 (0.6)	114 (0.5)	472 (0.3)
Renal Disease	589,473 (18.6)	8915 (29.9)	14,316 (30.5)	8729 (29.0)	5311 (24.6)	44,688 (27.4)
Congestive Heart Failure	1,130,525 (35.6)	15,778 (52.9)	27,190 (58.0)	17,992 (59.9)	12,371 (57.3)	78,235 (47.9)
Chronic Pulmonary Disease	84,295 (2.7)	29,828 (100)	46,903 (100)	30,055 (100)	21,579 (100)	163,342 (100)
Peripheral Vascular Disease	295,232 (9.3)	4536 (15.2)	8086 (17.2)	5106 (17.00)	3534 (16.4)	26,295 (16.1)
Cerebrovascular Disease	132,846 (4.2)	1705 (5.7)	2863 (6.1)	1778 (5.9)	1158 (5.4)	8427 (5.2)
Myocardial infarction	1,017,423 (32.1)	7493 (25.1)	11,685 (24.9)	7736 (25.7)	6498 (30.1)	51,840 (31.7)
Dementia	32,314 (1.0)	329 (1.1)	521 (1.1)	377 (1.3)	267 (1.2)	2290 (1.4)
Hemiplegia or Paraplegia	32,373 (1.0)	267 (0.9)	467 (1.0)	376 (1.3)	292 (1.4)	2229 (1.4)
Liver disease						
Mild	40,525 (1.3)	813 (2.7)	1256 (2.7)	828 (2.8)	561 (2.6)	2717 (1.7)
Moderate to severe	4605 (0.2)	72 (0.2)	125 (0.3)	72 (0.2)	51 (0.2)	288 (0.2)
Rheumatoid Disease	41,478 (1.3)	610 (2.1)	885 (1.9)	516 (1.7)	328 (1.5)	3,033 (1.9)
Peptic Ulcer Disease	8987 (0.3)	107 (0.4)	221 (0.5)	133 (0.4)	112 (0.5)	606 (0.4)
AIDS	2966 (0.1)	23 (0.1)	41 (0.1)	44 (0.2)	25 (0.1)	153 (0.1)
Outcome parameter						
In-hospital mortality—no. (%)	63,804 (2.0)	320 (1.1)	580 (1.2)	560 (1.9)	1,013 (4.7)	4,393 (2.7)
Hospital length of stay—d (IQR)	3 (1–6)	5 (3–9)	6 (3–10)	7 (4–12)	8 (4–14)	5 (2–8)
Peri- Interventional Ventilation Time—h (IQR) No. of ventilation support (%)	23 (5–92) N = 101,978 (3.2)	17.5 (5–59) N = 1122 (3.8)	17 (5–60.5) N = 2280 (4.9)	23 (7–76) N = 2385 (7.9)	35 (9–106) N = 3955 (18.3)	27 (7–95) N = 11,191 (6.9)

Characteristics of 3,464,369 hospitalized patients undergoing percutaneous coronary intervention the study cohort categorized by COPD severity.

XXX: For reasons of data protection, this number was not published.

In-hospital mortality increases with COPD severity grade from 1.1% in mild COPD (GOLD 1) to 4.7% in very severe COPD (GOLD 4). However, patients with an unspecified COPD grade showed also a high in-hospital mortality with 2.7%.

Patients with very severe COPD (GOLD 4) stayed longer in the hospital than patients with lower COPD severity grades (GOLD 4 to 1: 8 days (IQR, 4–14) vs. 7 days (IQR, 4–12) vs. 6 days (IQR, 3–10) vs. 5 days (IQR 3–9)). Patients with an unspecified COPD severity grade showed a HLOS of 5 days (IQR, 2–8).

The need for peri-interventional ventilation increases with COPD severity grade (3.8% vs. 4.9% vs. 7.9% vs. 18.3%). In addition, patients with very severe COPD (GOLD 4) presented a prolonged VT than patients with lower COPD severity grades (GOLD 4 to 1: 35 h (IQR, 9–106) vs. 23 h (IQR, 7–76) vs. 17 h (IQR, 5–60.5) vs. 17.5 h (IQR, 5–59)). Patients with an unspecified COPD severity grade showed in 6.9% the need for peri-interventional ventilation and a VT of 27 h (IQR, 7–95).

### Regression model analysis

Table 3 summarizes the key findings of the regression model analysis. The complete results are available as additional files (Additional files 2–8).

**Table 3:** Multivariable regression analysis.

	Mortality		Hospital length of stay		Ventilation time	
	Odds ratio (95% CI)	P-value	Coefficient (95% CI)	P-value	Coefficient (95% CI)	P-value
COPD	0.78 (0.76–0.81)	< 0.001	0.64 (0.62–0.67)	< 0.001	− 1.94 (− 4.34 to 0.43)	0.115
Severity of COPD						
Mild COPD (GOLD 1)	0.42 (0.37–0.48)	< 0.001	0.61 (0.55–0.68)	< 0.001	− 16.36 (− 21.92 to − 10.81)	< 0.001
Moderate COPD (GOLD 2)	0.46 (0.41–0.49)	< 0.001	1.13 (1.08–1.19)	< 0.001	− 19.47 (− 23.27 to − 15.66)	< 0.001
Severe COPD (GOLD 3)	0.57 (0.52–0.63)	< 0.001	1.71 (1.63–1.79)	< 0.001	− 9.10 (− 13.86 to − 4.33)	< 0.001
Very severe COPD (GOLD 4)	1.31 (1.23–1.42)	< 0.001	2.29 (2.18–2.39)	< 0.001	10.01 (5.14 to 14.88)	< 0.001
COPD, unspecified	0.94 (0.90–0.97)	0.001	0.24 (0.21–0.27)	< 0.001	2.67 (− 0.05 to 5.39)	0.054
COPD with exacerbation	1.46 (1.36–1.55)	< 0.001	2.93 (2.85–3.01)	< 0.001	− 4.21 (− 7.89 to − 0.53)	0.025
COPD with infection	2.37 (2.23–2.52)	< 0.001	4.99 (4.88–5.10)	< 0.001	19.00 (15.08 to 22.93)	< 0.001

Risk-Adjusted associations from multivariable regression analysis models analyzing the impact of COPD and COPD severity in 3,464,369 hospitalized patients undergoing percutaneous coronary intervention.

### Association between COPD and in-hospital mortality, HLOS and VT

COPD was associated with lower odds for in-hospital mortality (adjusted OR: 0.78, 95% CI: 0.76–0.81, p < 0.001). In addition, COPD was associated with a shorter HLOS (C: 0.64, 95% CI: 0.62–0.67, p < 0.001). The association between COPD and VT did not reach significance (C: − 1.94, 95% CI: − 4.34 to 0.43), p = 0.115).

### Association between COPD severity grades and in-hospital mortality, HLOS and VT

#### In-hospital mortality

A further regression model analysis demonstrated a lower risk of in-hospital mortality and mild COPD (GOLD 1, adjusted OR: 0.42, 95% CI: 0.37–0.48, p < 0.001), moderate COPD (GOLD 2, adjusted OR: 0.46, 95% CI: 0.41–0.49, p < 0.001) and severe COPD (GOLD 3, adjusted OR: 0.57, 95% CI: 0.52–0.63, p < 0.001) (Table 3). Only patients suffering from very severe COPD (GOLD 4) showed a higher risk of in-hospital mortality (adjusted OR: 1.31, 95% CI: 1.23–1.42, p < 0.001). Patients with an unspecified COPD severity grade showed a lower odds for in-hospital mortality (adjusted OR: 0.94, 95% CI: 0.90–0.97, p < 0.001).

In addition, patients suffering from COPD_e_ or COPD_i_ demonstrated a higher risk of in-hospital mortality (adjusted OR: 1.46, 95% CI: 1.36–1.55, p < 0.001 and adjusted OR: 2.37, 95% CI: 2.23–2.52, p < 0.001).

#### HLOS

Moreover, the further regression model analysis demonstrated a shorter HLOS in patients with mild COPD (C: 0.61, 95% CI: 0.55–0.68, p < 0.001). Patients suffering from moderate to very severe COPD showed a longer HLOS (GOLD 2, C: 1.13, 95% CI: 1.08–1.19, p < 0.001; GOLD 3, C: 1.71, 95% CI: 1.63–1.79, p < 0.001; GOLD 4, C: 2.29, 95% CI: 2.18–2.39, p < 0.001). Patients with an unspecified COPD severity grade showed a shorter HLOS (C: 0.24, 95% CI: 0.21–0.27, p < 0.001).

In addition, patients suffering from COPD_e_ or COPD_i_ demonstrated a longer HLOS (COPD_e_, C: 1.46, 95% CI: 1.36–1.55, p < 0.001 and COPD_i,_ C: 4.99, 95% CI: 4.88–5.10, p < 0.001).

#### VT

Additionally, the further regression model analysis demonstrated a shorter VT in patients with mild COPD (GOLD 1, C: − 16.36, 95% CI: − 21.92 to − 10.81, p < 0.001), moderate COPD (GOLD 2, C: − 19.47, 95% CI: − 23.27 to − 15.66, p < 0.001) and severe COPD (GOLD 3, C: − 9.10, 95% CI: − 13.86 to − 4.33, p < 0.001)). Only patients suffering from very severe COPD showed a prolonged VT (GOLD 4, C: 10.01, 95% CI: 5.14–14.88, p < 0.001). Patients with an unspecified COPD severity grade showed a prolonged VT (C: 2.67, 95% CI: − 0.05 to 5.39, p < 0.001).

In addition, patients suffering from COPD_i_ demonstrated a prolonged VT (C: 19.0, 95% CI: 15.08–22.93, p < 0.001). Patients suffering from COPD_e_ did not show a statistically significant difference in VT (C: − 4.21, 95% CI: − 7.89 to − 0.53, p = 0.025).

#### Sensitivity analysis to confirm the results

A sensitivity analysis excluding the group of unspecified COPD showed similar results, so that it must be assumed that the results are reliable (Additional file 8).

## Discussion

In this population-based retrospective cohort study, we investigated whether COPD, or certain grades of COPD severity, were associated with higher in-hospital mortality, HLOS and VT after PCI. The study population consisted of adult hospitalized patients undergoing PCI between 2015 and 2019 in Germany.

Our principal findings were a paradoxical association between COPD and in-hospital mortality as well as VT after PCI. Further regression model analysis demonstrated a paradoxical association between COPD and in-hospital mortality as well as VT after PCI in patients suffering from mild COPD to severe COPD. Only in patients with severe COPD, COPD_e_ and COPD_i,_ we found a higher risk for in-hospital mortality and a prolonged VT.

Various studies demonstrated the expected negative impact of COPD on mortality after PCI^8,12,25^. For example, a cohort study performed in Taiwan demonstrated an increased risk of hospital mortality, overall mortality, ischemic events as well as major adverse cardiac and cerebrovascular events in patients with COPD following PCI^12^. Furthermore, a systematic review and meta-analysis showed that major cardiac events and mortality were higher in patients suffering from COPD undergoing PCI^26^. In contrast, there are also studies that could not demonstrate a significant negative impact of COPD on in-hospital mortality or adverse cardiac events after PCI. Berger et al. showed that in-hospital major cardiac events, including death, did not differ between patients with and without COPD^19^. Other studies also failed to demonstrate higher in-hospital mortality in patients suffering from COPD compared to patients without COPD^20,27^. This contradicting background of evidence shows that the impact of COPD on PCI has not been investigated sufficiently. Therefore, we conducted a population-based retrospective cohort study.

In our study, we found a paradoxical association between COPD and in-hospital mortality. Similar unexpected results were reported from the impact of obesity on mortality for specific patient populations^28–30^. This phenomenon is also known as the “obesity paradox”. To explain this unexpected relationship, different approaches like non-proven causality in observational studies, confounding or different bias, exist^31^. In contrast to other studies, we investigated different COPD severity grades. Patients suffering from mild to severe COPD (GOLD 1 to 3) showed a paradoxical association between in-hospital mortality and VT. However, patients with very severe COPD (GOLD 4), COPD_e_ as well as COPD_i_ demonstrated a higher risk for in-hospital mortality. This result is consistent to other studies, e.g., the study of Lin et al. The authors found that patients with more frequent COPD exacerbations or recent hospitalized COPD exacerbation showed a higher risk for adverse events like overall mortality, ischemic events and major adverse cardiac and cerebrovascular events after PCI^12^.

The paradoxical association between mild to severe COPD (GOLD 1–3) and in-hospital mortality could be because clinicians are more alert with patients suffering from COPD. To prevent pulmonary deterioration, patients suffering from COPD may be forced to perform respiratory exercise or adequate upper body elevation to promote optimal gas exchange. In mild to severe COPD, these initiatives have a positive impact on the outcome. In patients suffering from very severe COPD (GOLD 4) or COPD_e_ and COPD_i_, these initiatives are probably no longer sufficiently effective due to the severity of the lung disease. Now, the expected negative association between COPD and in-hospital mortality becomes apparent. Our results implicate that clinicians should ensure optimal treatment of COPD to avoid a progression of the disease. An individual approach including treatment of comorbidities is certainly desirable, as Alter et al. showed that increased comorbidities were associated with COPD progression. Moreover, the authors demonstrated that differentiation between age- and COPD associated factors with impact on comorbidities is possible^32^. Likewise, Wang et al. demonstrated the increased morbidity of COPD patients by showing a correlation between LV geometric changes and systolic function impairment with FEV1^33^. In addition, our results show an adverse outcome when suffering from an acute exacerbation or infection. It would be favourable to avoid a PCI in this state of disease, if possible.

The unexpected paradoxical association between low to severe COPD (GOLD 1–3) and in-hospital mortality in patients undergoing PCI should be investigated in detail for several reasons. First, a paradoxical association between overweight or obese patients with COPD and mortality is already known^25,34,35^. Second, an “obesity paradox” could also be demonstrated in Japanese patients after PCI. Kaneko et al. showed that overweight and obesity was associated with better long-term outcome after PCI^36^. Based on these findings, an analysis of the body mass index our study population would be interesting. Perhaps, our study group of patients suffering from low to severe COPD is predominantly overweight or obese, which may support the “obesity paradox”. Third, Yamauchi et al.^34^ demonstrated that underweight COPD patients had a higher mortality compared to low- to normal-weight patients. Our results demonstrated that very severe COPD is associated with a higher risk of in-hospital mortality. Since very severe COPD is frequently associated with underweight, it would be interesting to estimate the body mass index of patients suffering from very severe COPD in our patient population.

Our study has various strengths. First, the cohort size: we analyzed more than 3 million cases, as all patients undergoing PCI in Germany between 2015 and 2019 were included. Second, we only analyzed data before the pandemic so that any influence of an undiagnosed lung affection by COVID-19 was deleted. Third, documentation of diagnosis and procedures can be considered as representative, as there is a requirement of documentation in Germany due to reimbursement based on a performance-based coding system. Nevertheless, “inaccurate” coding cannot be excluded completely, as seen in the relatively high number of patients in the unspecified COPD group (Tables 2 and 3). An inaccurate coding could be the result of the absence of lung function data, which is the main limitation of our study. A recently published meta-analysis demonstrated that undiagnosed and over-diagnosed COPD are common in primary healthcare. The authors demonstrated that COPD was not documented in 14–26% smokers with spirometry-confirmable COPD. In addition, they demonstrated that 25–50% of patients diagnosed with COPD did not have an airflow obstruction^37^. Other studies also revealed that the absence of spirometry is a common cause for under- or over-diagnosis of COPD^38,39^. In addition, large data analysis may be seen as a weakness, as significances will result more easily. Another weakness may be the inclusion of patients from a single country. Patients from other countries may be hospitalized shorter or longer compared to Germany. This could limit the generalisability of the results. In addition, readmissions to the hospital in the event of condition deterioration are not recorded in this approach and therefore, in-hospital mortality, HLOS and VT may be underestimated.

## Conclusions

In summary, this study shows a paradoxical association between mild to severe COPD (GOLD 1 to 3) and in-hospital mortality as well as VT, whereas very severe COPD (GOLD 4), COPD_e_ and COPD_i_ show a higher risk for in-hospital mortality. In addition, HLOS is longer in patients suffering from COPD, depending on the COPD severity grade.

To elucidate these results, prospective intervention studies should be conducted. Further research should illuminate long-term mortality including different countries and hospitalization periods. Moreover, body mass index should be included in future analysis to identify the impact of the “obesity paradox”.

### Supplementary Information

Supplementary Information.

## Data Availability

We designed an analysis protocol as a Stata do-file and the analysis of the Stata do-file was performed on the actual data by the Federal Statistical Office. The actual data are managed by the Federal Statistical Office. Results were returned to the authors after a detailed review and curation of the data to avoid a possible de-anonymization of individuals. All data published in this study are included in this article [and its supplementary information files].

## Abbreviations

COPD: Chronic obstructive pulmonary disease
PCI: Percutaneous coronary intervention
FEV1: Forced expiratory volume in 1 s
CAD: Coronary artery disease
COPDe: COPD exacerbation
COPDi: COPD infection
HLOS: Hospital length of stay
VT: Peri-interventional ventilation time
G-DRG: German Diagnosis-Related Groups
OPS: Operationen- und Prozedurenschlüssel
ICPM: International Classification of Procedures in Medicine
WHO: World Health Organization
IQR: Interquartile ranges
AUROC: Area under the receiver operating curve
RMSE: Root mean square error
CCI: Charlson Comorbidity Index
