# Supplementary material for: Association between chronic obstructive pulmonary disease and in-hospital mortality after percutaneous coronary intervention: a retrospective cohort study in Germany
Source: Sci Rep. 2024 Mar 13;14:6044. doi: 10.1038/s41598-024-56255-3 (PMC10933297; doi:10.1038/s41598-024-56255-3)
Supplement: Supplementary file 1 — Supplementary Information. [file 41598_2024_56255_MOESM1_ESM.pdf]

Additional file 1. Details of variable transcoding for procedures and diagnoses (OPS procedural code and ICD 10- classification).

**Percutaneous Coronary Intervention**	
**Percutaneous Coronary Intervention**	1275,1279a,305g0,305g1,33001, 8837k, 8837m, 8837p, 8837u, 8837v, 8837w, 883d0, 883d1, 883d2, 88370,88371,88372, 88375, 88376, 8837q, 8837t, 88399
**Chronic Obstructive Pulmonary Disease**	
Chronic Obstructive Pulmonary Disease I	J4483, J4493, J4403, J4413
Chronic Obstructive Pulmonary Disease II	J4482, J4492, J4402, J4412
Chronic Obstructive Pulmonary Disease III	J4481, J4491, J4401, J4411
Chronic Obstructive Pulmonary Disease IV	J4480, J4490, J4410, J4400
Chronic Obstructive Pulmonary Disease unspecified	J4489, J4499, J4409, J4419
Chronic Obstructive Pulmonary Disease with exacerbation	J441
Chronic Obstructive Pulmonary Disease with infection	J440
**Comorbidities (Charlson Comorbidity Index)**	
Acute Myocardial Infarction	I21, I22, I252
Congestive Heart Failure	I43, I50, I099, I110, I130, I132, I255, I420, I425, I426, I427, I428, I429, P290
Peripheral Vascular Disease	I70, I71, I731, I738, I739, I771, I790, I792, K551, K558, K559, Z958, Z959
Cerebrovascular Disease	G45, G46, I60, I61, I62, I63, I64, I65, I66, I67, I68, I69, H340
Dementia	F00, F01, F02, F03, G30, F051, G311
Chronic Pulmonary Disease	J40, J41, J42, J43, J44, J45, J46, J47, J60, J61, J62, J63, J64, J65, J66, J67, I278, I279, J684, J701, J703

Rheumatologic Disease	M05, M32, M33, M34, M06, M315, M351, M353, M360
Peptic Ulcer Disease	K25, K26, K27, K28
Mild Liver Disease	B18, K73, K74, K700, K701, K702, K703, K709, K713, K714, K715, K717, K760, K762, K763, K764, K768, K769, Z944
Moderate/Severe Liver Disease	K704, K711, K721, K729, K765, K766, K767, I850, I859, I864, I982
Diabetes without complications	E100, E101, E106, E108, E109, E110, E111, E116, E118, E119, E120, E121, E126, E128, E129, E130, E131, E136, E138, E139, E140, E141, E146, E148, E149
Diabetes with chronic complications	E102, E103, E104, E105, E107, E112, E113, E114, E115, E117, E122, E123, E124, E125, E127, E132, E133, E134, E135, E137, E142, E143, E144, E145, E147
Hemiplegia or Paraplegia	G81, G82, G041, G114, G801, G802, G830, G831, G832, G833, G834, G839
Renal Disease	N18, N19, Z49, N052, N053, N054, N055, N056, N057, N250, I120, I131, N032, N033, N034, N035, N036, N037, Z490, Z491, Z492, Z940, Z992
Cancer	C00, C01, C02, C03, C04, C05, C06, C07, C08, C09, C10, C11, C12, C13, C14, C15, C16, C17, C18, C19, C20, C21, C22, C23, C24, C25, C26, C30, C31, C32, C33, C34, C37, C38, C39, C40, C41, C43, C45, C46, C47, C48, C49, C50, C51, C52, C53, C54, C55, C56, C57, C58, C60, C61, C62, C63, C64, C65, C66, C67, C68, C69, C70, C71, C72, C73, C74, C75, C76, C81, C82, C83, C84, C85, C88, C90, C91, C92, C93, C94, C95, C96, C97

Metastatic Cancer	C77, C78, C79, C80
AIDS/HIV	B20, B21, B22, B23, B24, U60, U61, Z21

Additional file 2. Risk-Adjusted associations of **In-Hospital Mortality** from multivariable regression analysis models analysing the impact of COPD in 3,464,369 hospitalized patients undergoing percutaneous coronary intervention.

	Odds Ratio (95% CI)	P- Value
**COPD**	0.78 (0.76-0.81)	<0.001
**COPD with Exacerbation**	1.53 (1.43.1.63)	<0.001
**COPD with Infection**	2.53 (2.38-2.68)	<0.001
**Age**	1.04 (1.04-1.04)	<0.001
**Female**	0.99 (0.98-1.01)	0.532
**Emergency Hospital Admission**	2.95 (2.89-3.00)	<0.001
Charlson Comorbidity Score Items		
**Myocardial Infarction**	3.72 (3.66-3.79)	<0.001
**Chronic Heart Failure**	1.88 (1.85-1.91)	<0.001
**Peripheral Vascular Disease**	1.04 (1.02-1.07)	<0.001
**Cerebrovascular Disease**	1.50 (1.46-1.55)	<0.001
**Dementia**	1.64 (1.57-1.71)	<0.001
**Rheumatic Disease**	0.63 (0.58-0.68)	<0.001
**Peptic Ulcer Disease**	1.32 (1.19-1.35)	<0.001
**Mild Liver Disease**	1.27 (1.20-1.35)	<0.001
**Moderate to Severe Liver Disease**	5.24 (4.75-5.78)	<0.001
**Diabetes without Complications**	0.95 (0.93-0.96)	<0.001
**Diabetes with Complications**	1.14 (1.10-1.17)	<0.001
**Paraplegia or Hemiplegia**	1.86 (1.78-1.96)	<0.001

**Renal Disease**	0.91 (0.89-0.93)	<0.001
**Cancer**	1.91 (1.81-2.01)	<0.001
**Metastatic Cancer**	4.45 (4.13-4.79)	<0.001
**AIDS**	1.85 (1.50-2.28)	<0.001
Severity of Cardiac Catheterisation		
**Implantation of 1 Stent**	0.96 (0.94-0.98)	<0.001
**Implantation of 2 Stents**	1.17 (1.14-1.20)	<0.001
**Implantation of 3 Stents**	1.45 (1.42-1.50)	<0.001
**Implantation of 4 Stents**	1.70 (1.64-1.77)	<0.001
**Implantation of 5 Stents**	2.28 (2.15-2.40)	<0.001
**Implantation of >=6 Stents**	2.97 (2.77-3.16)	<0.001

Additional file 3. Risk-Adjusted associations of **Hospital Length of Stay** from multivariable regression analysis models analysing the impact of COPD in 3,464,369 hospitalized patients undergoing percutaneous coronary intervention.

	**Coefficient (95% CI)**	**P- Value**
**COPD**	0.64 (0.62-0.67)	<0.001
**COPD with Exacerbation**	3.30 (3.22-3.37)	<0.001
**COPD with Infection**	5.34 (5.24-5.45)	<0.001
**Age**	0.02 (0.02-0.02)	<0.001
**Female**	0.44 (0.45-0.46)	<0.001
**Emergency Hospital Admission**	2.45 (2.44-2.46)	<0.001
Charlson Comorbidity Score Items		
**Myocardial Infarction**	0.62 (0.61-0.63)	<0.001
**Chronic Heart Failure**	2.36 (2.35-2.37)	<0.001
**Peripheral Vascular Disease**	0.58 (0.56-0.60)	<0.001
**Cerebrovascular Disease**	1.84 (1.80-1.88)	<0.001
**Dementia**	2.74 (2.66-2.82)	<0.001
**Rheumatic Disease**	0.96 (0.90-1.01)	<0.001
**Peptic Ulcer Disease**	5.15 (4.98-5.32)	<0.001
**Mild Liver Disease**	2.60 (2.52-2.66)	<0.001
**Moderate to Severe Liver Disease**	6.44 (6.12-6.76)	<0.001
**Diabetes without Complications**	0.25 (0.23-0.26)	<0.001
**Diabetes with Complications**	1.19 (1.15-1.22)	<0.001
**Paraplegia or Hemiplegia**	3.26 (3.15-3.36)	<0.001

**Renal Disease**	1.33 (1.32-1.35)	<0.001
**Cancer**	2.57 (2.48-2.65)	<0.001
**Metastatic Cancer**	5.52 (5.31-5.74)	<0.001
**AIDS**	0.35 (0.15-0.55)	0.001
Severity of Cardiac Catheterisation		
**Implantation of 1 Stent**	-0.24 (-0.26- -0.23)	<0.001
**Implantation of 2 Stents**	0.06 (0.04-0.07)	<0.001
**Implantation of 3 Stents**	0.34 (0.32-0.36)	<0.001
**Implantation of 4 Stents**	0.67 (0.63-0.71)	<0.001
**Implantation of 5 Stents**	0.94 (0.87-1.01)	<0.001
**Implantation of >=6 Stents**	1.43 (1.33-1.53)	<0.001

Additional file 4. Risk-Adjusted associations of peri-interventional **Ventilation Time** from multivariable regression analysis models analysing the impact of COPD in 122,910 hospitalized patients undergoing percutaneous coronary intervention.

	Coefficient (95% CI)	P- Value
**COPD**	-1.94 (-4.34-0.43)	0.115
**COPD with Exacerbation**	-2.19 (-5.77-1.38)	0.229
**COPD with Infection**	21.00 (17.12-24.86)	<0.001
**Age**	-0.56 (-0.60- -0.51)	<0.001
**Female**	-11.04 (-12.26- -9.82)	<0.001
**Emergency Hospital Admission**	0.69 (-0.90- 2.27)	0.396
Charlson Comorbidity Score Items		
**Myocardial Infarction**	-6.39 (-7.79- -4.99)	<0.001
**Chronic Heart Failure**	1.96 (0.67-3.25)	0.003
**Peripheral Vascular Disease**	-4.47 (-6.23- -2.70)	<0.001
**Cerebrovascular Disease**	18.46 (15.74-21.18)	<0.001
**Dementia**	3.74 (0.06-7.41)	0.046
**Rheumatic Disease**	2.28 (-4.21-8.78)	0.491
**Peptic Ulcer Disease**	21.00 (12.75-29.24)	<0.001
**Mild Liver Disease**	15.91 (11.33-20.49)	<0.001
**Moderate to Severe Liver Disease**	28.89 (19.11-38.68)	<0.001
**Diabetes without Complications**	2.59 (1.21-3.97)	<0.001
**Diabetes with Complications**	4.62 (2.44-6.79)	<0.001
**Paraplegia or Hemiplegia**	17.93 (13.40-22.45)	<0.001

**Renal Disease**	-2.86 (-4.27- -1.44)	<0.001
**Cancer**	4.57 (0.18-9.00)	0.041
**Metastatic Cancer**	7.00 (-0.48-14.48)	0.067
**AIDS**	20.93 (2.51-39.34)	0.026
Severity of Cardiac Catheterisation		
**Implantation of 1 Stent**	5.78 (4.17.36)	<0.001
**Implantation of 2 Stents**	5.87 (4.08-7.67)	<0.001
**Implantation of 3 Stents**	4.73 (2.63-6.84)	<0.001
**Implantation of 4 Stents**	7.23 (4.22-10.24)	<0.001
**Implantation of 5 Stents**	8.19 (3.89-12.50)	<0.001
**Implantation of >=6 Stents**	8.38 (3.61-13.14)	0.001

Additional file 5. Risk-Adjusted associations of **In-Hospital Mortality** from multivariable regression analysis models analysing the impact of COPD and COPD severity in 3,464,369 hospitalized patients undergoing percutaneous coronary intervention.

	**Odds Ratio (95% CI)**	**P- Value**
	Severity of COPD	
**Mild COPD (GOLD 1)**	0.42 (0.37-0.48)	<0.001
**Moderate COPD (GOLD 2)**	0.46 (0.41-0.49)	<0.001
**Severe COPD (GOLD 3)**	0.57 (0.52-0.63)	<0.001
**Very severe COPD (GOLD 4)**	1.31 (1.23-1.42)	<0.001
**COPD with Exacerbation**	1.46 (1.36-1.55)	<0.001
**COPD with Infection**	2.37 (2.23-2.52)	<0.001
**COPD, unspecified**	0.94 (0.90-0.97)	0.001
**Age**	1.04 (1.04-1.04)	<0.001
**Female**	0.99 (0.98-1.01)	0.544
**Emergency Hospital Admission**	2.95 (2.89-3.00)	<0.001
	Charlson Comorbidity Score Items	
**Myocardial Infarction**	3.71 (3.64-3.78)	<0.001
**Chronic Heart Failure**	1.89 (1.86-1.92)	<0.001
**Peripheral Vascular Disease**	1.04 (1.02-1.07)	0.001
**Cerebrovascular Disease**	1.51 (1.46-1.55)	<0.001
**Dementia**	1.64 (1.57-1.71)	<0.001
**Rheumatic Disease**	0.63 (0.58-0.68)	<0.001
**Peptic Ulcer Disease**	1.32 (1.19-1.46)	<0.001

**Mild Liver Disease**	1.28 (1.21-1.36)	<0.001
**Moderate to Severe Liver Disease**	5.27 (4.77-5.81)	<0.001
**Diabetes without Complications**	0.95 (0.93-0.96)	<0.001
**Diabetes with Complications**	1.14 (1.10-1.17)	<0.001
**Paraplegia or Hemiplegia**	1.85 (1.77-1.94)	<0.001
**Renal Disease**	0.91 (0.90-0.93)	<0.001
**Cancer**	1.91 (1.82-2.01)	<0.001
**Metastatic Cancer**	4.46 (4.14-4.81)	<0.001
**AIDS**	1.85 (1.50-2.28)	<0.001
Severity of Cardiac Catheterisation		
**Implantation of 1 Stent**	0.96 (0.94-0.98)	<0.001
**Implantation of 2 Stents**	1.17 (1.14-1.20)	<0.001
**Implantation of 3 Stents**	1.46 (1.41-1.50)	<0.001
**Implantation of 4 Stents**	1.70 (1.64-1.77)	<0.001
**Implantation of 5 Stents**	2.27 (2.15-2.40)	<0.001
**Implantation of >=6 Stents**	2.97 (2.79-3.16)	<0.001

Additional file 6. Risk-Adjusted associations of **Hospital length of Stay** from multivariable regression analysis models analysing the impact of COPD and COPD severity in 3,464,369 hospitalized patients undergoing percutaneous coronary intervention.

	**Coefficient (95% CI)**	**P- Value**
	Severity of COPD	
**Mild COPD (GOLD 1)**	0.61 (0.55-0.68)	<0.001
**Moderate COPD (GOLD 2)**	1.13 (1.08-1.19)	<0.001
**Severe COPD (GOLD 3)**	1.71 (1.63-1.79)	<0.001
**Very severe COPD (GOLD 4)**	2.29 (2.18-2.39)	<0.001
**COPD with Exacerbation**	2.93 (2.85-3.01)	<0.001
**COPD with Infection**	4.99 (4.88-5.10)	<0.001
**COPD, unspecified**	0.24 (0.21-0.27)	<0.001
**Age**	0.02 (0.02-0.02)	<0.001
**Female**	0.45 (0.44-0.46)	<0.001
**Emergency Hospital Admission**	2.45 (2.44-2.46)	<0.001
	Charlson Comorbidity Score Items	
**Myocardial Infarction**	0.62 (0.61-0.64)	<0.001
**Chronic Heart Failure**	2.35 (2.34-2.36)	<0.001
**Peripheral Vascular Disease**	0.58 (0.56-0.60)	<0.001
**Cerebrovascular Disease**	1.84 (1.80-1.88)	<0.001
**Dementia**	2.74 (2.66-2.83)	<0.001
**Rheumatic Disease**	0.96 (0.90-1.02)	<0.001
**Peptic Ulcer Disease**	5.15 (4.98-5.32)	<0.001

**Mild Liver Disease**	2.58 (2.51-2.65)	<0.001
**Moderate to Severe Liver Disease**	6.43 (6.11-6.76)	<0.001
**Diabetes without Complications**	0.25 (0.24-0.26)	<0.001
**Diabetes with Complications**	1.19 (1.15-1.22)	<0.001
**Paraplegia or Hemiplegia**	3.27 (3.16-3.37)	<0.001
**Renal Disease**	1.33 (1.32-1.35)	<0.001
**Cancer**	2.55 (2.57-2.64)	<0.001
**Metastatic Cancer**	5.50 (5.29-5.72)	<0.001
**AIDS**	0.35 (0.15-0.55)	0.001
Severity of Cardiac Catheterisation		
**Implantation of 1 Stent**	-0,24 (-0,26- -0.23)	<0.001
**Implantation of 2 Stents**	0.06 (0.05-0.08)	<0.001
**Implantation of 3 Stents**	0.35 (0.32-0.37)	<0.001
**Implantation of 4 Stents**	0.68 (0.64-0.72)	<0.001
**Implantation of 5 Stents**	0.95 (0.88-1.02)	<0.001
**Implantation of >=6 Stents**	1.43 (1.34-1.53)	<0.001

Additional file 7: Risk-Adjusted associations of peri-interventional **Ventilation Time** from multivariable regression analysis models analysing the impact of COPD and COPD severity in 122,910 hospitalized patients undergoing percutaneous coronary intervention.

	**Coefficient (95% CI)**	**P- Value**
	Severity of COPD	
**Mild COPD (GOLD 1)**	-16.36 (-21.92- -10.81)	<0.001
**Moderate COPD (GOLD 2)**	-19.47 (-23.27- -15.66)	<0.001
**Severe COPD (GOLD 3)**	-9.10 (-13.86- -4.33)	<0.001
**Very severe COPD (GOLD 4)**	10.01 (5.14-14.88)	<0.001
**COPD with Exacerbation**	-4.21 (-7.89- -0.53)	0.025
**COPD with Infection**	19.00 (15.08-22.93)	<0.001
**COPD, unspecified**	2.67 (-0.05-5.39)	0.054
**Age**	-0.56 (-0.60—0.51)	<0.001
**Female**	-11.03 (-12.265- -9.81)	<0.001
**Emergency Hospital Admission**	0.70 (-0.88-2.28)	0.387
	Charlson Comorbidity Score Items	
**Myocardial Infarction**	-6.54 (-7.94- -5.14)	<0.001
**Chronic Heart Failure**	2.16 (0.87-3.45)	0.001
**Peripheral Vascular Disease**	-4.40 (-6.16- -2.64)	<0.001
**Cerebrovascular Disease**	18.54 (15.82-21.26)	<0.001
**Dementia**	3.74 (0.07-7.41)	0.046
**Rheumatic Disease**	2.32 (-4.17-8.81)	0.484
**Peptic Ulcer Disease**	20.82 (12.59-29.04)	<0.001

**Mild Liver Disease**	15.97 (11.40-20.54)	<0.001
**Moderate to Severe Liver Disease**	28.86 (19.08-38.64)	<0.001
**Diabetes without Complications**	2.60 (1.22-3.98)	<0.001
**Diabetes with Complications**	4.75 (2.58-6.93)	<0.001
**Paraplegia or Hemiplegia**	17.84 (13.31-22.36)	<0.001
**Renal Disease**	-2.75 (-4.17- -1.34)	<0.001
**Cancer**	4.67 (0.28-9.06)	0.037
**Metastatic Cancer**	7.14 (-0.32-14.60)	0.061
**AIDS**	20.91 (2.43-39.38)	0.027
Severity of Cardiac Catheterisation		
**Implantation of 1 Stent**	5.84 (4.25-7.42)	<0.001
**Implantation of 2 Stents**	5.91 (4.11-7.70)	<0.001
**Implantation of 3 Stents**	4.50 (2.70-6.90)	<0.001
**Implantation of 4 Stents**	7.23 (4.22-10.24)	<0.001
**Implantation of 5 Stents**	8.11 (3.81-12.42)	<0.001
**Implantation of >=6 Stents**	8.36 (3.61-13.12)	0.001

Additional file 8: Sensitivity analysis without unspecified COPD patients analysing the impact of COPD and COPD severity in 3,301,025 hospitalized patients undergoing percutaneous coronary intervention.

	Mortality		Hospital Length of Stay		Ventilation Time	
	**Odds Ratio (95% CI)**	**P-Value**	**Coefficient (95% CI)**	**P-Value**	**Coefficient (95% CI)**	**P-Value**
Severity of COPD						
**Mild COPD (GOLD 1)**	0.45 (0.40-0.5)	<0.001	0.66 (0.60-0.72)	<0.001	-15.92 (-21.60- -10.24)	<0.001
**Moderate COPD (GOLD 2)**	0.49 (0.44-0.53)	<0.001	1.20 (1.14-1.26)	<0.001	-18.92 (-23.25- -14.6)	<0.001
**Severe COPD (GOLD 3)**	0.65 (0.59-0.72)	<0.001	1.81 (1.73-1.89)	<0.001	-8.36 (-13.85- -2.88)	<0.001
**Very severe COPD (GOLD 4)**	1.54 (1.41-1.69)	<0.001	2.43 (2.32-2.54)	<0.001	10.90 (5.2-16.6)	<0.001
**COPD with Exacerbation**	1.14 (1.03-1.27)	<0.001	2.63 (2.53-2.74)	<0.001	-4.58 (-10.1- 0.94)	0.025
**COPD with Infection**	1.92 (1.74-2.12)	<0.001	4.79 (4.65-4.94)	<0.001	17.96 (12.17-23.74)	<0.001
